# Assessing recovery in treatment as usual provided by community child and adolescent mental health services

**DOI:** 10.1192/bjo.2021.44

**Published:** 2021-04-23

**Authors:** Naomi Gibbons, Emma Harrison, Paul Stallard

**Affiliations:** Child and Adolescent Mental Health Services, Oxford Health NHS Foundation Trust, Melksham Community Hospital, UK; Child and Adolescent Mental Health Services, Oxford Health NHS Foundation Trust, Melksham Community Hospital, UK; Child and Adolescent Mental Health Group, Department for Health, University of Bath, UK; and Child and Adolescent Mental Health Services, Oxford Health NHS Foundation Trust, UK

**Keywords:** Routine outcome measures, child and adolescent mental health services, recovery, reliable change, audit

## Abstract

**Background:**

Despite the importance of routinely assessing the outcomes of everyday practice, few studies have reported outcome metrics for child and adolescent mental health services (CAMHS).

**Aims:**

Our aim is to investigate reliable change and recovery rates for treatment as usual, provided by one community CAMHS over two time periods.

**Method:**

We prospectively audited accepted consecutive referrals from November 2017 to January 2018, and April to September 2019. Cases with paired outcomes were identified, and reliable change and recovery rates were calculated.

**Results:**

Baseline outcome data were obtained for 672 (78.2%) and 744 (77.5%) young people in 2018 and 2019, respectively. Of eligible participants, 174 (59.2%) and 155 (45.7%) completed at least one follow-up outcome measure in 2018 and 2019, respectively. Pre- and post-test scores on the Revised Child Anxiety and Depression Scale (RCADS) and Strengths and Difficulties Questionnaire (SDQ) showed a reduction in symptoms. Total RCADS scores showed 21–25% of participants reliably improved, with 44–49% showing reliable improvement on one or more subscale. On the SDQ, 11 (15.5%) and 19 (25.3%) participants reported reliable improvement on at least one subscale in 2018 and 2019, respectively. Reliable recovery rates ranged from 48 to 51% for youth-completed and 40 to 42% for parent-completed RCADS.

**Conclusions:**

Half of young people receiving treatment as usual from CAMHS reliably improved on at least one routine outcome measure subscale, improvement rates comparable with adult psychological therapies services. Our findings indicate that reliable change and recovery on subscale rather than total scores may be a better indication of outcomes.

There is increased focus on the use of outcome data to support improvements in clinical services providing interventions for children and young people with mental health problems.^1^ In the UK, data are reported nationally and are derived from routine outcome measures (ROMs) completed for cases seen within child and adolescent mental health services (CAMHS) on at least two occasions.^2^ The benefits of using ROMs include greater clinical responsiveness to the young person's progress, better engagement in therapy and more effective interventions.^3–5^ However, although routine outcome monitoring has recognised benefits, widespread uptake within CAMHS has been slow, with paired ROMs typically being obtained for <50% of cases.^6–8^ A recent evaluation of the caregiver-reported Strengths and Difficulties Questionnaire (SDQ), one of the core recommended outcome measures in the UK, revealed that although 40% were completed during assessment, only 3% were completed at 6-month follow-up.^9^

## Routine outcome monitoring

Although the routine use of ROMs is currently limited, even less is known about the outcomes that are achieved for young people receiving treatment as usual from CAMHS.^10^ Within everyday CAMHS, outcomes may not be consistent and may be lower than those obtained in well-conducted research studies, where interventions are delivered under more controlled conditions.^11^ In terms of outcome metrics, research studies typically report between-group (control versus treatment) differences, which is not particularly relevant or meaningful at an individual level.^12^ Instead, attention has turned to establishing reliable change,^13^ a metric used to determine whether significant change has occurred at an individual level and is not a result of measurement error.^14^ Additionally, reliable recovery explores reliable change for those within the clinical range at the start of treatment who then move to the normal range. It has been suggested that reliable change and recovery are more meaningful outcome metrics for patients attending everyday clinical services.^12^

## Reliable change in CAMHS

The adult Improving Access to Psychological Therapies (IAPT) programme aimed to achieve a 50% recovery rate, a target that was predicted to be lower in CAMHS because of the complexity and diversity of problems in young people accessing the service.^15^ ROM data collected from multiple services during the initial stages of the Children and Young Person IAPT programme have been reported. A national evaluation of paired data (*n* = 4464), collected between 2011 and 2015 from CAMHS in the UK, explored reliable recovery on the youth self-report Revised Child Anxiety and Depression Scale (RCADS), a commonly used ROM. Reliable recovery was 46% for those with anxiety, 42% for depression and 26% for those with comorbid anxiety and depressive disorders.^16^ Using a different outcome measure and rater, the parent-report version of the SDQ, a reliable recovery rate of 36% has been reported.^15,17^ These findings are consistent with a recent meta-analysis that found 40% of young people receiving treatment as usual from CAMHS reliably recovered, as assessed by a range of outcome measures.^10^ The review highlighted the dearth of research on the outcomes of treatment as usual and the considerable methodological shortfalls, and raised questions about the effectiveness of everyday CAMHS.^12^ This lack of published outcome data about CAMHS treatment as usual is concerning, particularly given the transformational aim of the Children and Young Person IAPT programme to embed ROMs within everyday services. It is unclear whether this objective has been achieved within individual services, or whether recovery rates have been maintained over time.

## Aims

Our aim is to contribute to the evidence base regarding the outcomes for children and adolescents attending everyday CAMHS in the UK. We will investigate reliable change and recovery rates, using paired outcome measures collected over two different time periods.

## Method

### Study design

We conducted in-depth prospective audits of new, consecutive referrals, accepted into Bath and North East Somerset, Swindon and Wiltshire community CAMHS during two separate time periods: November 2017 to January 2018 and April to September 2019. The time frames were pragmatically determined by the availability of psychology assistants to undertake the audits.

This study is an audit of anonymised collective data, and as such, ethical approval was not required. Patients provided informed consent to complete outcome measures and for this to be analysed.

### Outcome data

The service has a local procedure where ROMS are completed at baseline, after three sessions and at discharge. The service has mandated the use of the following core outcome measures, to be completed when considered appropriate:

- The Current View is a clinician-completed questionnaire that is conducted either on first contact with the patient or when there is a change in situation or understanding. It is used to provide an overview of presenting problems, problem severity, comorbidity, complexity (e.g. intellectual disability or parental health issues) and contextual factors (e.g. home or community), and impact on education/employment in terms of both attendance and attainment.^18,19^
- The RCADS is designed for children and young people aged 8–18 years, and is a 47-item questionnaire with subscales for major depression, panic disorder, obsessive–compulsive disorder, generalised anxiety, separation anxiety and social phobia. There is a youth self-report questionnaire and a parent-report version.^20^
- The SDQ is a 25-item questionnaire with subscales for emotional symptoms, conduct problems, hyperactivity/inattention, peer relationship problems and prosocial behaviour. It has self-report and parent-report versions.^21^

Clinicians choose the outcome measure they feel is most appropriate to assess the young person's problems. Essentially, the RCADS is primarily used for problems of anxiety and low mood, and the SDQ is primarily used for conduct or attention-deficit hyperactivity disorder presentations.

### Procedure

All accepted referrals during the two audit periods were identified through the Oxford Health National Health Service (NHS) Foundation Trust electronic patient information system. Records were screened to identify those who had completed the above outcome measures at assessment. Cases with baseline ROMs were reviewed to identify those young people who had been seen on more than three occasions. If ROMs were not available, the primary worker/care coordinator was prompted by email on three occasions and requested to complete follow-up ROMs. The second ROMs could therefore be completed at discharge or when still in treatment. These were either completed in paper format or inputted directly onto an online outcome monitoring system (True Colours for Windows).

### Data analysis

Baseline and follow-up scores for youth- and parent-report versions of the RCADS and SDQ were extracted and inputted into SPSS version 23 for Windows. The RCADS and SDQ consist of a number of subscales, which are used to inform the focus of the intervention and monitor progress. Subsequently, change may be specific to a particular subscale relating to the primary presenting problem rather than the total score. Therefore, we analysed baseline and follow-up data for each subscale.

Reliable change was calculated for each subscale of the RCADS and SDQ. First, we calculated Cronbach's alpha, using baseline data to assess the internal consistency of each scale. Second, we calculated the standard error of the difference score. Finally, we calculated the reliable change index (RCI) as 1.96 times the standard error of difference between pre- and post-test scores. Any change above or below the RCI is considered reliable at the 95% confidence level. The RCI was used to classify individuals as reliably improved, deteriorated or no change, on each subscale.

Recovery rates were calculated if there were sufficient numbers of paired outcomes and cases demonstrating reliable change. For the RCADS, raw scores were converted to age- and gender-adjusted *t*-scores. A *t*-score of ≥65 was used to classify young people as within the clinical range. For the SDQ, those with a total score placing them in the borderline or abnormal range were identified. Reliable recovery was determined if a young person above the clinical cut-off at baseline showed a reliable reduction in post-test scores and moved into the normal range.

## Results

### Assessment completion and baseline ROMS

The total number of accepted referrals identified in each audit was 1074 in 2018 and 1172 in 2019. For those seen where ROMS were judged to be appropriate, over three-quarters completed baseline measures (2018: *n* = 672, 78.2%; 2019: *n* = 744, 77.5%). Of those eligible for a follow-up ROM, 174 (59.2%) completed at least one questionnaire in 2018 and 155 (45.7%) completed at least one questionnaire in 2019. It is unclear why paired ROM completion rates were lower in 2019.

### Presentation, problem severity, comorbidity and complexity

Baseline clinician-completed Current View assessments were available for 464 (47.5%) of those assessed in 2018 and 310 (30.0%) of those assessed in 2019. The three most commonly identified problems across both of the years were generalised anxiety (69–70%), depression (65–67%) and social anxiety (59–68%). In 2018, 3506 separate problems were identified, with 39% (*n* = 1366) being rated as either moderate or severe. Although fewer problems were identified in 2019 (*n* = 2187,) more were rated as moderate or severe (45.3%, *n* = 990).

Comorbidity was high, with only 3% (*n* = 14) of referrals presenting with single problems in 2018 and 7.1% (*n* = 22) in 2019. The most common number of identified problems per referral was between five and ten (59.1% in 2018; 49.1% in 2019).

Across both audit periods, around a quarter of referrals occurred within a context of poor parental health. In 2019, a fifth of referrals had experienced abuse or neglect, and a sixth presented with pervasive developmental disorders.

### Pre- and post-test change

Paired outcome data on total internalising scores for the youth- and parent/carer-completed RCADS and total difficulties scores for the SDQ are summarised in Table 1. Average pre- and post-test scores on all measures completed by both young people and parents show a reduction (improvement) over time.

**Table 1:** Pre- and post-test youth- and parent-reported outcome data

Measure	Year	Pre mean (s.d.)	Post mean (s.d.)	*P*-value
RCADS young person	2018 (*n* = 145)	72.5 (23.9)	59.2 (25.9)	0.000*
	2019 (*n* = 123)	69.6 (26.9)	59.7 (29.8)	0.000*
RCADS parent/carer	2018 (*n* = 65)	63.8 (27.3)	51.8 (23.1)	0.001*
	2019 (*n* = 65)	63.0 (28.5)	55.0 (27.9)	0.057
SDQ young person	2018 (*n* = 71)	21.2 (6.2)	19.7 (6.4)	0.038*
	2019 (*n* = 75)	21.3 (3.9)	19.9 (4.8)	0.024*
SDQ parent/carer	2018 (*n* = 42)	19.0 (6.5)	17.55 (6.3)	0.121
	2019 (*n* = 37)	19.3 (7.7)	17.8 (8.1)	0.155
SDQ impact, young person	2018 (*n* = 70)	5.0 (3.3)	3.6 (2.8)	0.034*
	2019 (*n* = 69)	4.7 (2.3)	3.8 (3.1)	0.005*
SDQ impact, parent/carer	2018 (*n* = 40)	6.0 (3.0)	4.4 (3.0)	0.011*
	2019 (*n* = 31)	6.2 (2.7)	4.6 (3.4)	0.021*

RCADS, Revised Child Anxiety and Depression Scale; SDQ, Strengths and Difficulties Questionnaire.

* *P* < 0.05.

### Reliable change: youth-completed outcomes

Paired RCADS data was obtained for 145 young people in 2018 and 123 young people in 2019. Paired SDQ data was available for 71 young people in 2018 and 75 young people in 2019 (Table 2).

**Table 2:** Youth-reported reliable change

Youth-completed ROMs	Year	Reliable deterioration	No change	Reliably improved
Total RCADS	2018 (*n* = 145)	1 (0.7%)	114 (78.6%)	30 (20.7%)
	2019 (*n* = 132)	7 (5.7%)	86 (69.9%)	30 (24.4%)
RCADS – depression	2018 (*n* = 145)	7 (4.8%)	93 (64.2%)	45 (31.0%)
	2019 (*n* = 123)	16 (13.0%)	68 (55.3%)	39 (31.7%)
RCADS – separation anxiety	2018 (*n* = 145)	5 (3.4%)	116 (80.0%)	24 (16.6%)
	2019 (*n* = 123)	9 (7.3%)	91 (74.0%)	23 (18.7%)
RCADS – social phobia	2018 (*n* = 145)	5 (3.4%)	105 (72.4%)	35 (24.1%)
	2019 (*n* = 123)	8 (6.5%)	90 (73.2%)	25 (20.3%)
RCADS – generalised anxiety	2018 (*n* = 145)	5 (3.4%)	95 (65.5%)	45 (31.0%)
	2019 (*n* = 123)	8 (6.5%)	82 (66.7%)	33 (26.8%)
RCADS – obsessive–compulsive	2018 (*n* = 145)	5 (3.4%)	113 (77.9%)	27 (18.6%)
	2019 (*n* = 123)	7 (5.7%)	93 (75.6%)	23 (18.7%)
RCADS – panic	2018 (*n* = 145)	7 (4.8%)	110 (75.9%)	28 (19.3%)
	2019 (*n* = 123)	7 (5.7%)	85 (69.1%)	9 (25.2%)
Total SDQ	2018 (*n* = 71)	2 (2.8%)	58 (81.7%)	11 (15.5%)
	2019 (*n* = 75)	4 (5.3%)	52 (69.4%)	19 (25.3%)
SDQ – emotional	2018 (*n* = 71)	0 (0%)	62 (87.3%)	9 (12.7%)
	2019 (*n* = 75)	3 (4.0%)	62 (82.7%)	10 (13.3%)
SDQ – conduct	2018 (*n* = 71)	1 (1.4%)	68 (95.8%)	2 (2.8%)
	2019 (*n* = 75)	2 (2.7%)	69 (92%)	4 (5.3%)
SDQ – hyperactivity	2018 (*n* = 71)	1 (1.4%)	67 (94.4%)	3 (4.2%)
	2019 (*n* = 75)	4 (5.3%)	65 (86.7%)	6 (8.0%)
SDQ – peer	2018 (*n* = 71)	2 (2.8%)	65 (91.6%)	4 (5.6%)
	2019 (*n* = 75)	2 (2.7%)	69 (92%)	4 (5.3%)

ROMs, routine outcome measures; RCADS, Revised Child Anxiety and Depression Scale; SDQ, Strengths and Difficulties Questionnaire.

Overall, 69 (47.6%) young people in 2018 and 60 (48.8%) young people in 2019 reported reliable improvement on at least one subscale of the RCADS. Reliable improvement on the SDQ was more modest. Overall, 11 (15.5%) young people in 2018 and 19 (25.3%) young people in 2019 reported a reliable improvement on at least one subscale of the SDQ.

### Reliable change: parent/carer-completed outcomes

Paired RCADS were completed by 64 parents/carers in 2018 and by 65 parents/carers in 2019. Paired SDQs were completed by 42 parent/carers in 2018 and 37 parents/carers in 2019 (Table 3).

**Table 3:** Parent/carer-reported reliable change

Parent/carer-completed ROMs	Year	Reliable deterioration	No change	Reliably improved
Total RCADS	2018 (*n* = 64)	5 (7.8%)	45(70.3%)	14 (21.9%)
	2019 (*n* = 65)	4 (6.2%)	47 (72.3%)	14 (21.5%)
RCADS –depression	2018 (*n* = 64)	7 (10.9%)	43 (67.2%)	14 (21.9%)
	2019 (*n* = 65)	6 (9.2%)	41 (63.1%)	18 (27.7%)
RCADS – separation anxiety	2018 (*n* = 64)	4 (6.2%)	49 (76.6%)	11 (17.2%)
	2019 (*n* = 65)	2 (3.1%)	46 (70.8%)	17 (26.2%)
RCADS – social anxiety	2018 (*n* = 64)	5 (7.8%)	45 (70.3%)	14 (21.9%)
	2019 (*n* = 65)	3 (4.6%)	53 (81.5%)	9 (13.8%)
RCADS – generalised anxiety	2018 (*n* = 64)	4 (6.2%)	50 (78.1%)	10 (15.6%)
	2019 (*n* = 65)	9 (13.8%)	40 (61.5%)	16 (24.6%)
RCADS – obsessive–compulsive	2018 (*n* = 64)	4 (6.2%)	50 (78.1%)	10 (15.6%)
	2019 (*n* = 65)	7 (10.8%)	46 (70.8%)	12 (18.5%)
RCADS – panic	2018 (*n* = 64)	1(1.6%)	50 (78.1%)	13 (20.3%)
	2019 (*n* = 65)	5 (7.7%)	46 (70.8%)	14 (21.5%)
Total SDQ	2018 (*n* = 42)	1 (2.4%)	30 (71.4%)	11 (26.2%)
	2019 (*n* = 37)	5 (13.5%)	22 (59.5%)	10 (27.0%)
SDQ – emotional	2018 (*n* = 42)	2 (4.8%)	38 (90.4%)	2 (4.8%)
	2019 (*n* = 37)	0 (0%)	30 (81.1%)	7 (18.9%)
SDQ – conduct	2018 (*n* = 42)	2 (4.8%)	38 (90.4%)	2 (4.8%)
	2019 (*n* = 37)	0 (0%)	35 (94.6%)	2 (5.4%)
SDQ – hyperactivity	2018 (*n* = 42)	1 (2.4%)	39 (92.8%)	2 (4.8%)
	2019 (*n* = 37)	5 (13.5%)	30 (81.1%)	2 (5.4%)
SDQ – peer	2018 (*n* = 42)	0 (0%)	36 (85.7%)	6 (14.3%)
	2019 (*n* = 37)	2 (5.4%)	32 (86.5%)	3 (8.1%)

ROMs, routine outcome measures; RCADS, Revised Child Anxiety and Depression Scale; SDQ, Strengths and Difficulties Questionnaire.

In both years, 29 parents/carers (45%) reported that their child had reliably improved on at least one subscale of the RCADS. As with the youth self-reported SDQ, there was comparatively less reliable change over time. In 2018, 11 parents (26.2%) reported a reliable improvement on at least one subscale, with a similar rate in 2019 (*n* = 10; 27%).

### Recovery

Given the comparatively small number of paired SDQs and the number who demonstrated a reliable improvement (range 11–19, depending on year and rater), we did not undertake any further analysis of recovery on this measure. For the RCADS, almost 90% of those who completed baseline assessments scored within the clinical range. Follow-up RCADS assessments highlight that half of the young people assessed reported reliable recovery on at least one subscale of the RCADS, compared with 40% of those assessed by parents (Table 4).

**Table 4:** Youth- and parent/carer-reported recovery

	2018	2019
Youth-rated recovery and reliable recovery		
Number of paired RCADS	145	123
Number in borderline or clinical range at baseline	129 (89.0%)	109 (88.6%)
Number recovered at follow-up (moved to normal range)	85 (65.9%)	67 (61.5%)
Number reliably recovered at follow-up	62 (48.1%)	56 (51.4%)
Parent/carer-rated recovery and reliable recovery	2018	2019
Number of paired RCADS	64	65
Number in borderline or clinical rage at baseline	57 (89.1%)	61 (93.8%)
Number recovered at follow-up (moved to normal range)	33 (57.9%)	30 (46.2%)
Number reliably recovered at follow-up	24 (42.1%)	24 (39.3%)

RCADS, Revised Child Anxiety and Depression Scale.

## Discussion

The aim of this study was to determine the outcomes of treatment as usual provided by one community CAMHS over time. We used different outcome metrics of significant change, reliable change and recovery, and explored young person and parent/carers ratings on two commonly used ROMs.

Our cohorts presented with predominantly anxiety and/or mood disorders, and as such, we had more paired RCADS than SDQs. Data from the Current View assessment support Wolpert et al's assertions that CAMHS referrals are often complex and comorbid, and present within a challenging context.^14^ Pre- and post-test total scores showed a reduction (improvement) in youth- and parent-completed ratings over time, which was particularly marked on the RCADS and the impact assessment of the SDQ. Reliable change and recovery on individual subscales revealed improvement rates of up to 45–48% on the RCADS and up to 25–27% on the SDQ, depending on the informant. Given the predominance of emotional disorders in our cohort, the RCADS may be a more sensitive outcome measure. The SDQ was developed as a behavioural screening questionnaire, and although its brevity is a strength, only five items assess emotional symptoms.^22^ Similarly, agreement between informants on the same measure vary, and correlations are often low.^23^ Young people may be more aware of changes in internal mood states or cognitions that are not directly observable or noticed by parents/carers. These findings highlight the importance of ensuring that outcome measures reflect the primary presenting problems, and of carefully choosing the primary informant.

The reliable recovery rates we report in this predominantly comorbid, emotionally disordered cohort are higher than those reported by others, and are more in line with recovery rates reported in the IAPT programme for working-age adults in the UK.^16,10,24,25^ This may reflect our analysis of individual subscales rather than total scores. The use of subscales has been advocated as an important aspect of routine clinical practice, where relevant subscales are recommended to be completed session by session, to track progress.^26^ The subscale will reflect the primary problem and the focus of the intervention. The outcome of an intervention for social anxiety, for example, may be better reflected on the social anxiety subscale of the RCADS, and may become diluted if change is assessed solely on total scores. This view is supported by an evaluation of a specialist obsessive–compulsive disorder service that found broad outcome measures seriously underestimated the effectiveness of the service.^27^ Our findings suggest that assessing change on specific subscales, rather than broad total scores, may be a more appropriate way of assessing outcomes. Clinically, this will involve a discussion with the young person to agree what outcomes or subscales are important for them.^10^

Although the reliable recovery rates we report are encouraging, it is evident that half of the cohort showed no reliable improvement. There is also the possibility that some may have reliably deteriorated, although rates of deterioration were low (2018: 0.7%; 2019: 5.7%). There is clearly a need to improve the effectiveness of treatment as usual, and to find ways to help the sizeable proportion of young people who are not currently responding to the interventions they are receiving. A greater emphasis on supervised practice may be helpful, and the routine use of outcome monitoring can alert clinicians to patients who are not responding to treatment, which can be discussed during clinical supervision.^12^ It is also important to acknowledge that clinical change and recovery may not be the only important outcomes of a CAMHS intervention. Stabilisation and prevention of deterioration are also important indicators of a successful outcome, and need to be acknowledged.^28^

The comparison of outcomes over different audit periods enables trends in presentations and outcomes to be identified. For patients, contemporary, meaningful and relevant local information about recovery and reliable change can help with decision-making. For clinicians the routine provision of this data may help to allay any suspicions or negativity, and promote a more positive attitude toward the routine use of outcomes.^2^ At a service level, changes in presentations and outcomes can be quickly identified, and any necessary training or supervision implemented. Finally, with the increased national focus on the outcomes of CAMHS, repeated outcome audits provide additional in-depth, local contextual data that can help commissioners understand the outcomes that are achieved in everyday practice.^1^ This can lead to further exploration of issues such as problem severity, chronicity and diagnosis on outcomes, and how current interventions may need to be modified to become more effective.

### Strengths and limitations

These two audits undertaken at different time periods provide consistent and encouraging results. Nonetheless, our study has a number of limitations that need to be acknowledged. First, although we report paired outcome data from almost 400 informants, we recognise that our sample is small. The number of paired SDQs we were able to analyse was particularly small. As such, our evaluation of the SDQ lacks power and our results may not be sensitive to change. Second, we obtained paired outcome data for between 46–60% of eligible young people, and so our findings may not be representative of those who receive treatment from CAMHS. Some cases without paired outcome data may have been dissatisfied or dropped out of the service, leading to our change rates being overinflated. Similarly, our findings may underrepresent recovery. Although we report paired ROMs, we do not know whether the second ROMs were completed at the end of the intervention or whether it was still in progress. Third, although we report reliable change and recovery, we do not know whether this is attributable to the treatment as usual the young people received from CAMHS. Spontaneous remission rates in mental health are high, and improvements may occur without any intervention. Fourth, although we were able to analyse paired outcomes for approximately half of eligible cases, there is still a significant amount of missing data. It is important to increase the number of paired ROMs to fully understand the effects of the service. In this study, we prompted clinicians to undertake the second ROMs, but we do not have data to determine whether such an approach increased completion rates. Finally, this study was undertaken within one mental healthcare trust. Although the service is based on the ithrve model, an integrated, person centred and needs led approach for delivering mental health services for children, young people and their families, recommended in the NHS Long Term Plan (NHS 2019),^29^ there may be important differences in service organisation or provision that limit the generalisability of our findings

To conclude, the results of these two in-depth audits are consistent, and detail the encouraging outcomes obtained from treatment as usual provided by community CAMHS. Reliable change and recovery rates indicate that up to half of those for whom data was available improved on at least one subscale. Our findings highlight the importance of agreeing the primary outcome and informant at the onset of the intervention. Furthermore, attention should be paid to specific subscale change, which may better reflect the focus of the intervention provided.

## Data Availability

The data that support the findings of this study are available on request from the corresponding author, P.S. The data are not publicly available due to restrictions.

## References

[1] NHS England. Five-Year Forward View. NHS England, 2014 (https://www.england.nhs.uk/wp-content/uploads/2014/10/5yfv-web.pdf).

[2] Hall C, Moldavsky M, Baldwin L, Marriott M, Newell K, Taylor J, The use of routine outcome measures in two child and adolescent mental health services: a completed audit cycle. BMC Psychiatry 2013; 13: 270. 10.1186/1471-244X-13-270 PMC4015925 24139139

[3] Bickman L, Kelley S, Breda C, de Andrade A, Riemer M. Effects of routine feedback to clinicians on mental health outcomes of youths: results of a randomized trial. Psychiatr Serv 2011; 62(12): 1423–9. 22193788 10.1176/appi.ps.002052011

[4] Bickman L, Douglas S, De Andrade A, Tomlinson M, Gleacher A, Olin S, Implementing a measurement feedback system: a tale of two sites. Adm Policy Ment Health 2016; 43(3): 410–25. 25876736 10.1007/s10488-015-0647-8 PMC4608853

[5] Unsworth G, Cowie H, Green A. Therapists’ and clients’ perceptions of routine outcome measurement in the NHS: a qualitative study. Couns Psychother Res 2012; 12(1): 71–80.

[6] Baruch G, Vrouva I. Collecting routine outcome data in a psychotherapy community clinic for young people: findings from an ongoing study. Child Adolesc Ment Health 2010; 15(1): 30–6. 32847206 10.1111/j.1475-3588.2009.00531.x

[7] Batty M, Moldavsky M, Foroushani P, Pass S, Marriott M, Sayal K, Implementing routine outcome measures in child and adolescent mental health services: from present to future practice. Child Adolesc Ment Health 2013; 18(2): 82–7. 32847291 10.1111/j.1475-3588.2012.00658.x

[8] Hall C, Moldavsky M, Taylor J, Sayal K, Marriott M, Batty M, Implementation of routine outcome measurement in child and adolescent mental health services in the United Kingdom: a critical perspective. Eur Child Adolesc Psychiatry 2014; 23(4): 239–42. 23896764 10.1007/s00787-013-0454-2 PMC3973864

[9] Morris A, Macdonald A, Moghraby O, Stringaris A, Hayes R, Simonoff E, Sociodemographic factors associated with routine outcome monitoring: a historical cohort study of 28,382 young people accessing child and adolescent mental health services. Child Adolesc Ment Health 2021; 26(1): 56–64. 32544982 10.1111/camh.12396

[10] Bear H, Edbrooke-Childs J, Norton S, Krause K, Wolpert M. Systematic review and meta-analysis: outcomes of routine specialist mental health care for young people with depression and/or anxiety. J Am Acad Child Adolesc Psychiatry 2020; 59(7): 810–41. 31881268 10.1016/j.jaac.2019.12.002

[11] Weisz J, Kuppens S, Ng M, Eckshtain D, Ugueto A, Vaughn-Coaxum R, What five decades of research tells us about the effects of youth psychological therapy: a multilevel meta-analysis and implications for science and practice. Am Psychol 2017; 72(2): 79–117. 28221063 10.1037/a0040360

[12] Walkup J, Parkhurst J, Lavigne J. Editorial: promoting quality psychotherapy: it is not the process but the outcome that matters! J Am Acad Child Adolesc Psychiatry 2020; 59(7): 797–9. 32439400 10.1016/j.jaac.2020.05.004

[13] Jacobson N, Follette W, Revenstorf D. Psychotherapy outcome research: methods for reporting variability and evaluating clinical significance. Behav Therapy 1984; 15(4): 336–52.

[14] Wolpert M, Görzig A, Deighton J, Fugard A, Newman R, Ford T. Comparison of indices of clinically meaningful change in child and adolescent mental health services: difference scores, reliable change, crossing clinical thresholds and ‘added value’ - an exploration using parent rated scores on the SDQ. Child Adolesc Ment Health 2015; 20(2): 94–101. 32680384 10.1111/camh.12080

[15] Wolpert M, Jacob J, Napoleone E, Whale A, Calderon A, Edbrooke-Childs J. Child- and Parent-Reported Outcomes and Experience from Child and Young People's Mental Health Services 2011–2015. Child Outcomes Research Consortium, 2016 (https://www.corc.uk.net/media/1544/0505207_corc-report_for-web.pdf).

[16] Edbrooke-Childs J, Wolpert M, Zamperoni V, Napoleone E, Bear H. Evaluation of reliable improvement rates in depression and anxiety at the end of treatment in adolescents. BJPsych Open 2018; 4(4): 250–5. 29998818 10.1192/bjo.2018.31 PMC6060492

[17] Fugard A, Stapley E, Ford T, Law D, Wolpert M, York A. Analysing and reporting UK CAMHS outcomes: an application of funnel plots. Child Adolesc Ment Health 2014; 20(3): 155–62. 32680403 10.1111/camh.12086

[18] Jones M, Hopkins K, Kyrke-Smith R, Davies R, Vostanis P, Wolpert M. Current View Tool Completion Guide. CAMHS Press, 2013 (https://www.ucl.ac.uk/evidence-based-practice-unit/sites/evidence-based-practice-unit/files/pub_and_resources_resources_for_profs_current_view.pdf).

[19] Vostanis P, Martin P, Davies R, De Francesco D, Jones M, Sweeting R, Development of a framework for prospective payment for child mental health services. J Health Serv Res Policy 2015; 20(4): 202–9. 25899484 10.1177/1355819615580868

[20] Chorpita B, Yim L, Moffitt C, Umemoto L, Francis S. Assessment of symptoms of DSM-IV anxiety and depression in children: a Revised Child Anxiety and Depression Scale. Behav Res Ther 2000; 38(8): 835–55. 10937431 10.1016/s0005-7967(99)00130-8

[21] Goodman R. The Strengths and Difficulties Questionnaire: a research note. J Child Psychol Psychiatry 1997; 38(5): 581–6. 9255702 10.1111/j.1469-7610.1997.tb01545.x

[22] Goodman R, Meltzer H, Bailey V. The Strengths and Difficulties Questionnaire: a pilot study on the validity of the self-report version. Eur Child Adolesc Psychiatry 1998; 7(3): 125–30. 9826298 10.1007/s007870050057

[23] Goodman R. Psychometric properties of the Strengths and Difficulties Questionnaire. J Am Acad Child Adoles Psychiatry 2001; 40(11): 1337–45. 10.1097/00004583-200111000-00015 11699809

[24] Clark D, Canvin L, Green J, Layard R, Pilling S, Janecka M. Transparency about the outcomes of mental health services (IAPT approach): an analysis of public data. Lancet 2018; 391: 679–86. 29224931 10.1016/S0140-6736(17)32133-5 PMC5820411

[25] Gyani A, Shafran R, Layard R, Clark DM. Enhancing recovery rates: lessons from year one of IAPT. Behav Res Ther 2013; 51(9): 597–606. 23872702 10.1016/j.brat.2013.06.004 PMC3776229

[26] Law D, Wolpert M, eds. Guide to Using Outcomes and Feedback Tools with Children, Young People and Families (2nd edn). CAMHS Press, 2014 (https://www.ucl.ac.uk/evidence-based-practice-unit/sites/evidence-based-practice-unit/files/pub_and_resources_resources_for_profs_guide_to-using.pdf).

[27] Lee W, Jones L, Goodman R, Heyman I. Broad outcome measures may underestimate effectiveness: an instrument comparison study. Child Adolesc Ment Health 2005; 10(3): 143–4. 32806851 10.1111/j.1475-3588.2005.00350.x

[28] Wallis P, Potier J, Milson G, Beck A. Delivering psychological services for children and young people with emotional and behavioural difficulties, and their families, in specialist CAMHS settings. Digested 2015; 48: 48–59.

[29] NHS. The NHS Long Term Plan. NHS, 2019 (https://www.longtermplan.nhs.uk/).

